# Nutritional Impacts of *Arthrospira platensis* Nanoparticles on Growth, Intestinal and Liver Histology, Antioxidant Status, and Immunological Response in Mullet (*Mugil cephalus*)

**DOI:** 10.1155/anu/6028970

**Published:** 2026-07-23

**Authors:** Ahmed I. A. Mansour, Mohamed Ashour, Mohamed M. Mabrouk, Ahmed F. Abdelhamid, Othman F. Abdelzaher, Roshmon Thomas Mathew, Abdallah Tageldein Mansour, Elsayed S. I. Mohammed, Yousef Ahmed Alkhamis, Michelyne Haroun, Einar Ringø, Eman Y. Mohammady

**Affiliations:** ^1^ Aquaculture Division, National Institute of Oceanography and Fisheries (NIOF), Cairo, Egypt, azhar.edu.eg; ^2^ Fish Resources Research Center, King Faisal University, Hofuf-420, Al-Ahsa 31982, Saudi Arabia, kfu.edu.sa; ^3^ Fish Production Department, Faculty of Agriculture, Al-Azhar University, Cairo 11823, Egypt, azhar.edu.eg; ^4^ Zoology Department, Faculty of Science, Al-Azhar University, Cairo, Egypt, azhar.edu.eg; ^5^ Animal and Fish Production Department, College of Agricultural and Food Sciences, King Faisal University, Al-Ahsa, Saudi Arabia, kfu.edu.sa; ^6^ Avian Research Center, King Faisal University, Hofuf-420, Al-Ahsa 31982, Saudi Arabia, kfu.edu.sa; ^7^ Faculty of Biosciences, Fisheries and Economics, Norwegian College of Fishery Science, UiT The Arctic University of Norway, Tromsø, Norway, uit.no

**Keywords:** antioxidant enzymes, *Arthrospira platensis*, growth performance, immune response, *Mugil cephalus*, nanoparticles

## Abstract

This study investigates the impact of dietary supplementation with nanoparticles of *Arthrospira platensis* (previously known as *Spirulina platensis*) on the performance of the mullet *Mugil cephalus* during a 63‐day feeding trial. Fish (14.75 ± 0.23 g/fish) were randomly distributed into five groups of 30 fish per hapa, with three replicates per group. Five experimental diets were prepared to assess nano‐*A. platensis* supplementation at three inclusion levels: 0.5, 5, and 10 g/kg (SN_0.05%_, SN_0.5%_, and SN_1%_). Two control groups were included for comparison: a negative control without additives (S_0%_) and a positive control with 1% non‐nanoform *A. platensis* (S_1%_). Among all SN groups, the fish fed the SN_0.05%_ diet showed significant (*p* ≤ 0.05) values for final weight (FW), specific growth rate (SGR), weight gain (WG), feed conversion ratio (FCR), protein efficiency ratio (PER), and feed efficiency ratio (FER). Also, the middle intestine showed marked improvements (*p* ≤ 0.05) in villus height and width, muscularis mucosa thickness, and the number of goblet cells when fish were fed the various SN levels or the S_1%_ diet. Moreover, fish treated with *A. platensis* nanoparticles showed a noticeable enhancement in hepatic histomorphology, with the SN_0.05%_ group exhibiting the most intact hepatic structure. However, the S_0%_ diet had the highest alanine aminotransferase (ALT) and aspartate aminotransferase (AST) compared to other diets supplemented with different *A. platensis* levels. No significant difference (*p* > 0.05) in albumin levels was observed. Non‐nano *A. platensis* improved immunoglobulin M (IgM) activity compared with the control (S_0%_). The nanoform diets resulted in the highest lysozyme (LYZ) activity, with the SN_0.05%_ treatment showing the highest values of all groups. Fish fed *A. platensis*, either as nanoparticles or in its non‐nano form, showed significantly lower triglyceride and cholesterol levels (*p* ≤ 0.05) than those that were fed the control diet (S_0%_). Additionally, all diets containing either nano‐ or non‐nanoform *A. platensis* (SN_0.05%_, SN_0.5%_, SN_1%_, and S_1%_) resulted in increased activities of glutathione peroxidase (GPx), superoxide dismutase (SOD), and catalase (CAT), alongside decreased malondialdehyde (MDA) levels, in comparison to S_0_%. In general, our findings showed that *A. platensis* nanoparticles improve *M. cephalus* growth performance, intestinal and hepatic histomorphology, immune response, feed utilization, and antioxidant enzyme activity. Therefore, nano‐*A. platensis* could be an efficient feed additive for *M. cephalus*, making mullet farming more environmentally friendly and cost‐effective.

## 1. Introduction

Aquaculture is an important sector of the fishery industry, which has expanded rapidly in parallel with population growth. This expansion has required the adoption of intensive production systems to meet the increasing demand for fish as a source of lean meat [1, 2]. Recently, intensive aquaculture production has faced several challenges. The higher feed requirements, disease susceptibility, and impaired immune function in cultured fish are the most common problems [3]. As a result, feed formulation has emerged as a key strategy for improving gut function and digestion efficiency and increasing the abundance of beneficial microbiota under intensive production conditions [4].

Antibiotics and vaccines have been widely used in disease control in aquaculture. However, antibiotic use has raised serious concerns due to its association with immunosuppression and food safety risks [5, 6]. The use of antibiotics in aquafeeds has been banned in several countries due to antibiotic residues, which may cause antimicrobial resistance [7]. Thus, it is essential to find sustainable nutritional strategies that enhance disease resistance, increase growth rate, and minimize environmental impact.

Functional feed additives have been recommended as practical substitutes for antibiotics. Among these additives, the microalga *Arthrospira platensis* (*A. platensis*) has been suggested as a promising candidate [8, 9]. It is characterized by a high nutritional value, including proteins, essential minerals, vitamins, polyunsaturated fatty acids, polysaccharides, and bioactive pigments. These components may play a crucial role in supporting metabolic efficiency and immunomodulation in fish [10, 11].

Dietary *A. platensis* has the ability to improve the growth performance and feed utilization in several cultured fish species under intensive production conditions. Its bioactive ingredients are linked to enhanced intestinal morphology, increased digestive activity, and modulation of innate and adaptive immune responses [11, 12]. It also contains potent antioxidant pigments that effectively neutralize reactive oxygen species, protect cells from injury, and maintain cellular integrity [13].

Improving the efficiency of functional feed additives through nanoparticle formulation can increase nutrient bioavailability and physiological efficiency [14, 15]. Conventional *A. platensis* supplementation has shown beneficial impacts on several fish species [16–18]. In its nanoparticle form, *A. platensis* has enhanced the growth performance of Nile tilapia (*Oreochromis niloticus*) [19].

Evaluating *A. platensis* nanoparticles as a feed additive for commercially important fish species is crucial for advancing aquaculture production. Among those fish species, *Mugil cephalus* (flathead grey mullet) is an essential species in Egypt and other countries that practice aquaculture. Mullet is valued for its high market demand, adaptability to marine, brackish, and freshwater systems, and efficient utilization of low‐trophic‐level feeds [20–23]. It is also highly responsive to dietary quality, making it an appropriate model for evaluating advanced nutritional strategies.

While the effects of conventional *A. platensis* supplementation have been reported in several studies, the impacts of its nano‐formulation as a feed supplement in *M. cephalus* remain poorly understood. This gap makes it hard for readers to know whether nano‐formulation can help this commercially valuable fish species reach its full metabolic and immunological potential. Consequently, the present study examines the effects of incorporating nano‐*A. platensis* on the antioxidative capacity, growth performance, and immune responses of *M. cephalus*.

## 2. Materials and Methods

### 2.1. Diets and Design

**Figure 1:**
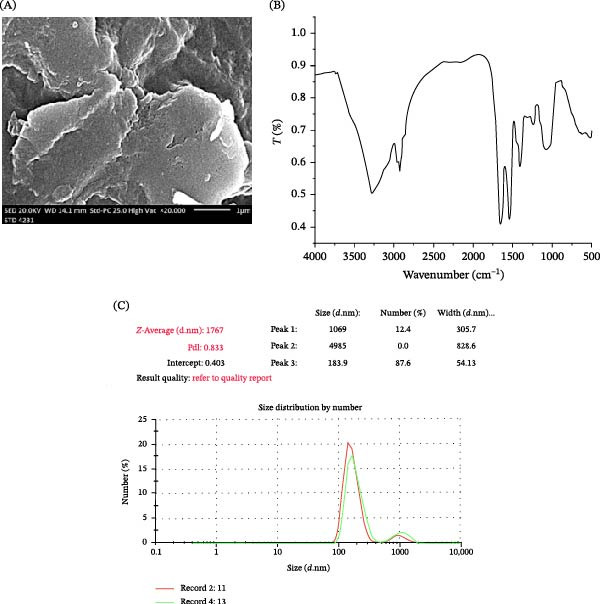
Characterization of *A. platensis* nanoparticles, based on our previously published data: (A) scanning electron microscope, (B) zeta potential, and (C) FTIR [25, 26].

In this work, five experimental diets (groups) were prepared (Table 1) to examine the effects of *Arthrospira platensis* NIOF17/003 nanoparticles on fish performance for 63 days. The experiment was designed to test nano‐*A*. *platensis* supplementation by incorporating it into the diets at three different inclusion levels: 0.5, 5, and 10 g/kg (SN_0.05%_, SN_0.5%_, and SN_1%_). To allow proper comparison, two control treatments were included in the experimental design: a negative control diet without any additives (S_0%_) and a positive control diet supplemented with 1% *A. platensis* in its non‐nanoform (S_1%_). *A. platensis* NIOF17/003 was obtained from El‐Khadra Lake, a saline‐alkaline habitat in Wadi El‐Natrun, northwest Egypt. The strain was genetically confirmed and registered in GenBank under Accession Number MW396472, as documented by Mabrouk et al. [14]. The *A. platensis* nanoparticles were prepared at the Egyptian Petroleum Research Institute (EPRI) in Nasr City, Cairo. Using a planetary ball mill (PM 400, four grinding stations), the biomass was processed to obtain nanoparticles with an average size of 183.9 nm, following the procedure outlined in our previous work [25]. Based on our previously published data, the characterization (scanning electron microscopy, zeta potential, and FTIR) of *A. platensis* nanoparticles has been performed as presented in Figure 1 [25, 26].

**Table 1:** Ingredients and experimental diets profile (g/kg diet, dry matter).

Ingredients (%)	S_0_%	Nano‐*A. platensis* (%)			S_1_%
		SN_0.05_%	SN_0.5_%	SN_1_%	
Fishmeal	100	100	100	100	100
Soybean meal	380	380	380	380	380
Corn gluten	70	70	70	70	70
Yellow corn	240	240	240	240	240
Wheat bran	150	150	150	150	150
Vitamin and mineral mix^a^	15	15	15	15	15
Vitamin C	5	5	5	5	5
Soybean oil	40	40	40	40	40
Non‐nanoform *A. platensis* (S)	0	0	0	0	10
Nano‐*A. platensis* (NS)	0	0.5	5	10	0
Proximate analysis					
Crude protein	310.00	314.00	312.50	314.60	312.50
Crude fat	68.30	67.10	68.50	68.80	68.50
Ash	52.90	53.20	53.10	53.70	53.10
Nitrogen‐free extract^b^	515.90	512.80	513.0	510.0	513.0
Gross energy^c^ (MJ/kg diet)	18.88	18.90	18.90	18.89	18.90

*Note:* S_0%_: negative control diet contains 0 g *A. platensis*/kg. SN_0.05%_, SN_0.5%_, and SN_1%_: diets enriched with *A. platensis* nanoparticles at concentrations of 0.5, 5, and 10 g/kg of feed, respectively. S_1%_: positive control diet enriched with non‐nanoform *A. platensis* at a concentration of 10 g/kg.

^a^Vitamin and mineral mixture kg^−1^ of mixture contains: 4800 I.U. Vit A, 2400 IU cholecalciferol (vit. D), 40 g Vit E, 8 g Vit K, 4.0 g Vit B12, 4.0 g Vit B2, 6 g Vit B6, 4.0 g, pantothenic acid, 8.0 g nicotinic acid, 400 mg folic acid, 20 mg biotin, 200 g choline, 4 g copper, 0.4 g iodine, 12 g iron, 22 g manganese, 22 g zinc, 0.04 g selenium, folic acid, 1.2 mg; niacin, 12 mg; d‐calcium pantothenate, 26 mg; pyridoxine, HCl, 6 mg; riboflavin, 7.2 mg; thiamin, HCl, 1.2 mg; sodium chloride (NaCl, 39% Na, 61% Cl), 3077 mg; ferrous sulfate (FeSO_4_·7H_2_O, 20% Fe), 65 mg; manganese sulfate (MnSO_4_, 36% Mn), 89 mg; zinc sulfate (ZnSO_4_·7H_2_O, 40% Zn), 150 mg; copper sulfate (CuSO_4_·5H_2_O, 25% Cu), 28 mg; potassium iodide (KI, 24% K, 76% I).

^b^Nitrogen free extract = 100−(crude protein + lipid + ash + fiber content).

^c^Gross energy calculated using gross calorific values of 23.63, 39.52, and 17.15 kJg^−1^ for protein, fat, and carbohydrate, respectively, according to Brett [24].

The diet’s biochemical profile is detailed in Table 1, following the AOAC (2012) guidelines. The dietary constituents were ground in a feed grinder until a homogeneous mixture was achieved (Hobart Corporation, Troy, OH, USA) and subsequently thoroughly combined with *A. platensis* (either in non‐nano form or SN) and sunflower oil, as previously described [14]. In brief, distilled water was incorporated into the premixed ingredients, and the resulting mixture was thoroughly homogenized until a fluffy substance was obtained. The dough was molded into granules employing a manual noodle machine. The moist pellets were dried at ambient temperature and then stored at 4°C until use.

### 2.2. Fish Cultivation

All techniques employed in this study were conducted in accordance with the pertinent guidelines and regulations established by the National Institute of Oceanography and Fisheries (NIOF). Grey mullet (*Mugil cephalus*) were obtained from the Rosita Fry Collection Center (RFCC) on the Mediterranean Sea Coast, Al‐Behira, Egypt. Upon arrival at the Fish Research Station, located at the Baltim, Alexandria Branch of NIOF, Egypt, fish were acclimated for 2 weeks in a cement pond (4 × 2 × 1.25 m^3^). During this period, commercial feed (31% crude protein) was fed at 3% of body weight, split into three daily meals. After acclimation, 450 fish (averaging 14.75 ± 0.23 g) were randomly placed into 15 net haps (70 cm × 70 cm × 100 cm) in concrete ponds (400 cm × 200 cm × 100 cm), with 30 fish per hapa to represent five treatments and three replicates for a 63‐day trial. Fish were weighed twice a week to adjust feeding rates, and water quality was monitored throughout the experimental period. The daily feed ration was set at 3% of total biomass and offered in three equal meals at 09:00, 11:00, and 15:00 h. Water samples were collected regularly to measure the dissolved oxygen, temperature, pH, ammonia, nitrate, and nitrite concentrations following the protocol described by APHA [27]. All parameters remained within acceptable ranges for fish culture.

### 2.3. Intestinal and Hepatic Histomorphometry Examination

Liver and intestinal tissues from three fish per replicate were collected, fixed, and processed for routine histopathological examination [28]. Microscopic examination was used to assess the tissue structure, and measurements of villus width and height and muscularis mucosa thickness were quantified for statistical analysis [29]

### 2.4. Serum Biochemical Assay

Blood samples were collected from the caudal vein of three sedated fish for each replication. Serum was analyzed for liver enzymes, specifically aspartate aminotransferase (AST) and alanine aminotransferase (ALT), and protein profile (total protein, albumin, and globulin) [30–33], as well as lipid profile (triglycerides and cholesterol) and urea [34–36].

### 2.5. Nonspecific Immune Response Markers

Activities of immunoglobulin M (IgM) and lysozyme (LYZ) were performed using Bio‐Diagnostic kits (Giza, Egypt) according to the procedures described by Ai and Anderson [37] and Ellis [38], respectively.

### 2.6. Hepatic Antioxidant Activity

Liver samples from five fish per replicate were collected after anesthesia with clove oil (10 mg/L), homogenized, and rinsed in ice‐cold phosphate buffer (pH 7.0). After centrifugation (3000 × g for 10 min), the supernatant was used to determine antioxidant and oxidative stress markers. Superoxide dismutase (SOD) and catalase (CAT) activities were measured following established protocols [39, 40], while malondialdehyde (MDA) and glutathione peroxidase (GPx) levels were assessed as described by Moin [41] and Dogru et al. [42] approaches, respectively.

### 2.7. Data Collection, Calculations, and Statistical Analysis

At the end of the experimental period, the growth performance and feed efficiency parameters were determined using the following equations:

Specific growth rate SGR%/day

Feed conversion ratio FCR=Total consumed feed WG

Feed efficiency ratio FER=WG gFeed intake g

Protein efficiency ratio PER=WG g Protein intake g.

Prior to statistical analysis, data were examined for normality and homogeneity of variance using Levene’s test, and percentage data were subjected to arcsine transformation [43]. Treatment effects were evaluated using one‐way ANOVA, and mean comparisons were performed using Duncan’s multiple range test software [44] with SPSS. A *p*‐value of ≤ 0.05 was deemed statistically significant, and all data were presented as mean ± standard deviation (SD). Figures were produced using GraphPad Prism 8 [45].

## 3. Results

### 3.1. Growth Performance Indices

Mullet *M. cephalus* growth and nutrient utilization of fed varying levels of nano‐form *A. platensis* (SN_0.05%_, SN_0.5%_, and SN_1%_), alongside the control diet (S_0%_) and the non‐nanoform *A. platensis* diet (S_1%_), are shown in Table 2. Fish‐fed SN_0.05%_, SN_0.5%_, and SN_1%_ vary significantly (*p* ≤ 0.05) from fish fed S_0%_ and S_1%_. Among all SN groups (Table 2), the fish‐fed group SN_0.05%_ revealed significant results (*p* ≤ 0.05) for final weight (FW), feed conversion ratio (FCR), weight gain (WG), specific growth rate (SGR), protein efficiency ratio (PER), and feed efficiency ratio (FER).

**Table 2:** Growth performance and feed utilization indices of mullet *M. cephalus* fed experimental diets.

Indices	S_0%_	Nano‐*A. platensis* (%)			S_1%_	*p*‐Value
		SN_0.05%_	SN_0.5%_	SN_1%_		
IW	14.80 ± 0.05^a^	14.57 ± 0.40^a^	14.63 ± 0.50^a^	14.96 ± 0.10^a^	14.80 ± 0.30^a^	0.608^ns^
FW	29.89 ± 1.61^d^	39.44 ± 1.09^a^	37.64 ± 1.15^ab^	36.66 ± 0.32^b^	34.44 ± 0.94^c^	0.001
WG	15.09 ± 1.71^d^	24.87 ± 0.93^a^	23.01 ± 1.21^ab^	21.70 ± 0.21^b^	19.64 ± 0.99^c^	0.001
SGR	1.12 ± 0.04^d^	1.58 ± 0.03^a^	1.50 ± 0.02^ab^	1.42 ± 0.01^bc^	1.34 ± 0.02^c^	0.001
FCR	2.29 ± 0.36^a^	1.37 ± 0.04^c^	1.46 ± 0.06^c^	1.55 ± 0.01^bc^	1.86 ± 0.08^b^	0.003
FER	0.44 ± 0.08^d^	0.73 ± 0.02^a^	0.69 ± 0.03^ab^	0.65 ± 0.00^b^	0.54 ± 0.03^c^	0.004
PER	1.46 ± 0.25^d^	2.43 ± 0.06^a^	2.28 ± 0.10^ab^	2.15 ± 0.01^b^	1.79 ± 0.08^c^	0.002

*Note:* S_0%_: negative control diet contains 0 g *A. platensis*/kg. S_1%_: positive control diet enriched with non‐nanoform *A. platensis* at a concentration of 10 g/kg. SN_0.05%_, SN_0.5%_, and SN_1%_: diets enriched with *A. platensis* nanoparticles at concentrations of 0.5, 5, and 10 g/kg of feed, respectively. Data are presented as mean ± SD (*n* = 3 replicates per treatment). Means within the same row bearing different lowercase superscript letters (a, b, c, d) indicate statistically significant differences among treatments based on one‐way ANOVA, followed by Duncan’s multiple range test. ns = nonsignificant (*p* > 0.05).

Abbreviations: FCR, feed conversion ratio; FER, feed efficiency ratio; FW, final weight (g); PER, protein efficiency ratio; SGR, specific growth rate (SGR, %/day); WG, weight gain (g).

**Figure 2:**
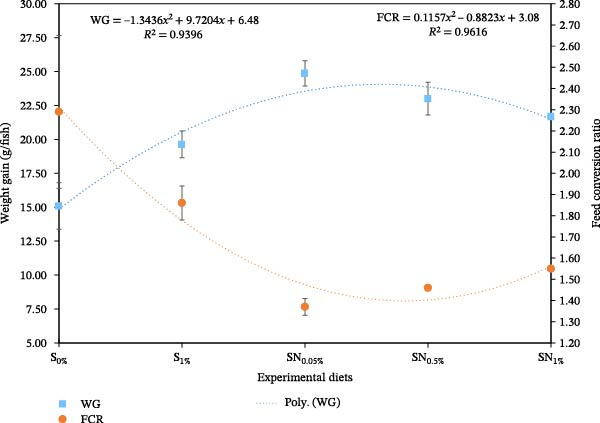
Polynomial regression between weight gain and feed conversion ratio of mullet *Mugil cephalus* fed the experimental diets for 63 days. S_0_%: Negative control diet contains 0 g *A. platensis*/kg. S_1_%: Positive control diet enriched with non‐nanoform *A. platensis* at a concentration of 10 g/ kg. SN_0.05_%, SN_0.5_%, and SN_1_%: Diets enriched with *A. platensis* nanoparticles at concentrations of 0.5, 5, and 10 g/kg of feed, respectively. The quadratic regression model indicates a highly significant relationship (*p* < 0.001) with a high coefficient of determination (R^2^ = 0.9616 and 0.9396 for FCR and WG, respectively).

The fish WG and FCR polynomial regression model (PRM) are shown in Figure 2. The results reported that the PRM for FCR considerably decreased (*R* ^2^ = 0.742) and the PRM for WG considerably enhanced (*R* ^2^ = 0.8506) in the SN_0.05%_ group (0.5 g SNP/ kg diet). This result suggests that the ideal *A. platensis* nanoparticle level is 0.5 g/kg diet, as determined by the PRM (Figure 2).

### 3.2. Intestinal and Hepatic Histomorphometry

The middle intestine showed marked increases (*p* ≤ 0.05) in villus width and height, muscularis mucosa thickness, and the number of goblet cells when fish were fed the various SN levels or the S_1%_ diet (Table 3). Fish that received the diet enriched with 0.05% NS showed the highest VW, VL, GC, and MM thickness values.

**Table 3:** Intestinal histomorphometry of mullet, *M. cephalus* fed the experimental diets.

Parameter	S_0%_	Nano‐*A. platensis* (%)			S_1%_	*p*‐Value
		SN_0.05%_	SN_0.5%_	SN_1%_		
VL (μm)	171.4 ± 4.30^e^	230.30 ± 3.10^a^	224.30 ± 1.70^b^	207.30 ± 1.90^c^	196.4 ± 2.80^d^	0.001
VW (μm)	64.10 ± 1.20^d^	77.10 ± 0.55^a^	72.30 ± 0.95^bc^	73.80 ± 0.90^b^	71.20 ± 0.30^c^	0.001
MMT (μm)	43.40 ± 0.60^d^	49.10 ± 0.60^a^	48.40 ± 0.40^b^	46.90 ± 0.34^b^	44.20 ± 0.50^c^	0.001
GC (No.)	54.40 ± 1.10^e^	72.10 ± 0.60^a^	67.60 ± 0.80^b^	62.20 ± 0.50^c^	58.90 ± 0.30^d^	0.001

*Note:* S_0%_: negative control diet contains 0 g *A. platensis*/kg. S_1%_: positive control diet enriched with non‐nanoform *A. platensis* at a concentration of 10 g/kg. SN_0.05%_, SN_0.5%_, and SN_1%_: diets enriched with *A. platensis* nanoparticles at concentrations of 0.5, 5, and 10 g/kg of feed, respectively. Data are presented as mean ± SD (*n* = 3 replicates per treatment). Means within the same row bearing different lowercase superscript letters (a, b, c, d, e) indicate statistically significant differences among treatments based on one‐way ANOVA (*p* < 0.001 for VL, VW, MMT, and GC), followed by Duncan’s multiple range test.

Abbreviations: GC, goblet cells (No.); MMT, muscularis mucosa thickness (μm); VL, villus length (μm); VW, villus width (μm).

**Figure 3:**
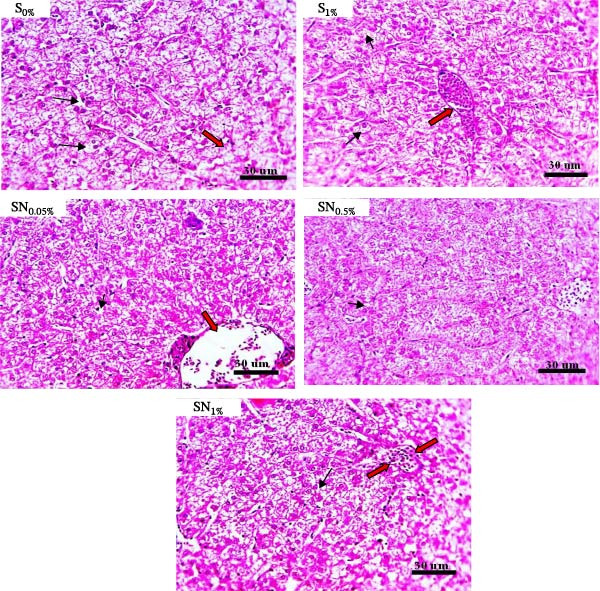
Hepatic histomorphometry of *M. cephalus* fed different levels of *A. platensis*. H&E, bar = 30 μm. Data showing hepatic lipid vacuoles, dilated blood vessels, and hemorrhage. Scale bar = 30 μm. S_0%_: negative control diet contains 0 g *A. platensis*/kg. SN_0.05%_, SN_0.5%_, and SN_1%_: diets enriched with *A. platensis* nanoparticles at concentrations of 0.5, 5, and 10 g/kg of feed, respectively. S_1%_: positive control diet enriched with non‐nanoform *A. platensis* at a concentration of 10 g/kg. Black arrow: lipid vacuoles; red arrow: vascular dilation and congestion.

Moreover, fish treated with *A. platensis* nanoparticles showed a noticeable enhancement in liver morphology, with the SN_0.05%_ group exhibiting the most intact hepatic structure and the least lipid vacuolation among all treatments (Figure 3). Also, the SN_0.5%_ and SN_1%_ groups exhibited reduced lipid accumulation and less vascular dilation. Fish fed the S_1%_ diet exhibited some improvement in the liver structure, but the changes were not as marked as those seen in the groups receiving the nanoparticle‐supplemented diets. Overall, the SN_0.05%_ level produced the most marked hepatoprotective effect (Figure 3).

### 3.3. Serum Biochemical Indices

**Figure 4:**
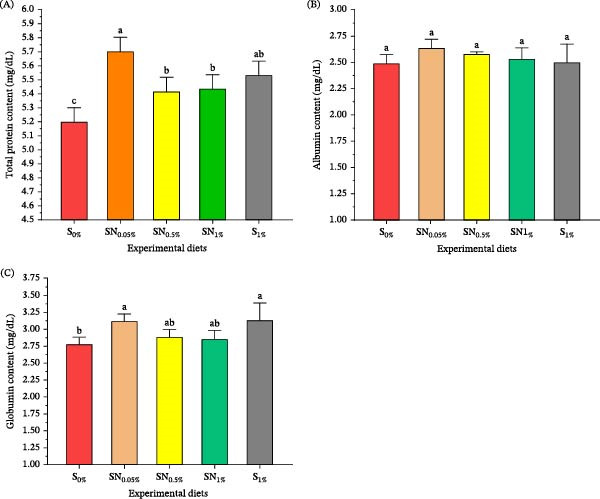
Serum biochemical indices of total protein (A), albumin (B), and globulin (C) contents of mullet *M. cephalus* fed the experimental diets. S_0%_: negative control diet contains 0 g *A. platensis*/kg. SN_0.05%_, SN_0.5%_, and SN_1%_: diets enriched with *A. platensis* nanoparticles at concentrations of 0.5, 5, and 10 g/kg of feed, respectively. S_1%_: positive control diet enriched with non‐nanoform *A. platensis* at a concentration of 10 g/kg. Data are presented as mean ± SD (*n* = 3 replicates per treatment). Means with different letters differ significantly among treatments based on one‐way ANOVA (*p* < 0.004 for total protein; *p* = 0.476 ns for albumin; *p* = 0.067 ns for globulin), followed by Duncan’s multiple range test for total protein only.

Figure 4 shows the TP, ALB, and GLU contents of mullet fed the experimental diets. Compared with all experimental diets, fish‐fed SN_0.05%_ recorded a significantly (*p* ≤ 0.05) improved value of TP content (Figure 4A), followed by S_1%_, SNP_0.5%_, and SN_1%_, while the lowest result was reported by fish‐fed S_0%_ (negative control group). For ALB content, no significant (*p* > 0.05) differences were obtained from all experimental diets (Figure 4B). For GLU content (Figure 4C), groups fed the SN_0.05%_ and S_1%_ diets recorded the highest significant (*p* ≤ 0.05) values, followed by SN_0.5%_ and SN_1%_, while the lowest was observed in those fed the S_0%_ diet.

**Figure 5:**
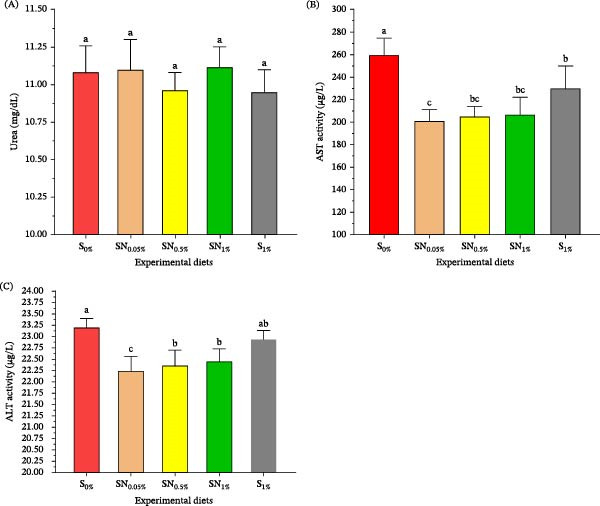
Urea (A), aspartate aminotransferase (AST) (B), and alanine aminotransferase (ALT) activities (C) of mullet *M. cephalus* fed the experimental diets. S_0%_: negative control diet contains 0 g *A. platensis*/kg. SN_0.05%_, SN_0.5%_, and SN_1%_: diets enriched with *A. platensis* nanoparticles at concentrations of 0.5, 5, and 10 g/kg of feed, respectively. S_1%_: positive control diet enriched with non‐nanoform *A. platensis* at a concentration of 10 g/kg. Data are presented as mean ± SD (*n* = 3 replicates per treatment). Means with different letters differ significantly among treatments based on one‐way ANOVA (*p* = 0.756 ns for urea; *p* < 0.002 for AST; *p* < 0.006 for ALT), followed by Duncan’s multiple range test for AST and ALT.

However, the urea levels and the AST and ALT values of fish‐fed experimental diets are shown in Figure 5. Regarding urea value, no significant (*p* > 0.05) difference was observed between all experimental groups (Figure 5A). Compared to S_0%_ and S_1%_, fish‐fed *A. platensis* nanoparticle groups (SN_0.05%_, SN_0.5%_, and SN_1%_) showed a lower significant (*p* ≤ 0.05) value for AST and ALT activities (Figure 5B,C). Among all fish‐fed SN groups, the lowest significant values of AST and ALT were reported by fish‐fed SN_0.05%_.

### 3.4. Nonspecific Immune Responses and Lipid Profile

**Figure 6:**
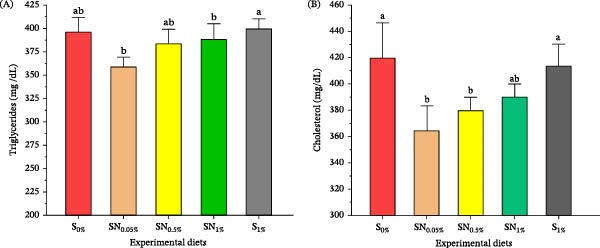
Triglyceride (A) and cholesterol (B) concentrations of *M. cephalus* fed experimental diets. S_0%_: negative control diet contains 0 g *A. platensis*/kg. SN_0.05%_, SN_0.5%_, and SN_1%_: diets enriched with *A. platensis* nanoparticles at concentrations of 0.5, 5, and 10 g/kg of feed, respectively. S_1%_: positive control diet enriched with non‐nanoform *A. platensis* at a concentration of 10 g/kg. Data are presented as mean ± SD (*n* = 3 replicates per treatment). Means with different letters differ significantly among treatments based on one‐way ANOVA (*p* < 0.030 for triglyceride; *p* < 0.014 for cholesterol), followed by Duncan’s multiple range test for both triglyceride and cholesterol.

Figure 6A,B displays lipid profiles, including triglyceride and cholesterol contents of fish‐fed different experimental diets, respectively. Fish fed *A. platensis*, either as nanoparticles or in its non‐nanoform, showed significantly lower triglyceride and cholesterol levels (*p* ≤ 0.05) than those fed the control (S_0%_). SN_0.05%_ exhibited the most pronounced reduction (*p* ≤ 0.05). Overall, the nanoform led to greater decreases in triglycerides and cholesterol than the non‐nanoform (S_1%_).

**Figure 7:**
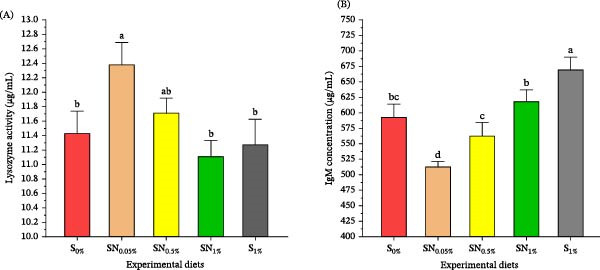
Nonspecific immune responses, including lysozyme activity (A) and immunoglobulin M (IgM) concentration (B), of *M. cephalus* fed the experimental diets. S_0%_: negative control diet contains 0 g *A. platensis*/kg. SN_0.05%_, SN_0.5%_, and SN_1%_: diets enriched with *A. platensis* nanoparticles at concentrations of 0.5, 5, and 10 g/kg of feed, respectively. S_1%_: positive control diet enriched with non‐nanoform *A. platensis* at a concentration of 10 g/kg. Data are presented as mean ± SD (*n* = 3 replicates per treatment). Means with different letters differ significantly among treatments based on one‐way ANOVA (*p* < 0.001 for lysozyme; *p* < 0.001 for IgM), followed by Duncan’s multiple range test for both lysozyme and IgM.

Regarding immune responses, particularly LYZ and IgM activities, groups fed diets enriched with *A. platensis*, whether in nano‐ or non‐nanoform, exhibited increased LYZ and IgM activities compared to the control group (S_0%_) (Figure 7A,B). The SN nanoparticle groups (SN_0.05%_, SN_0.5%_, and SN_1%_) showed significantly (*p* ≤ 0.05) LYZ and IgM levels higher than S_0%_ and S_1%_. Among the nanoparticle treatments, SN_0.05%_ exhibited the highest significant increase in both LYZ and IgM activities.

### 3.5. Hepatic Antioxidant Response

**Figure 8:**
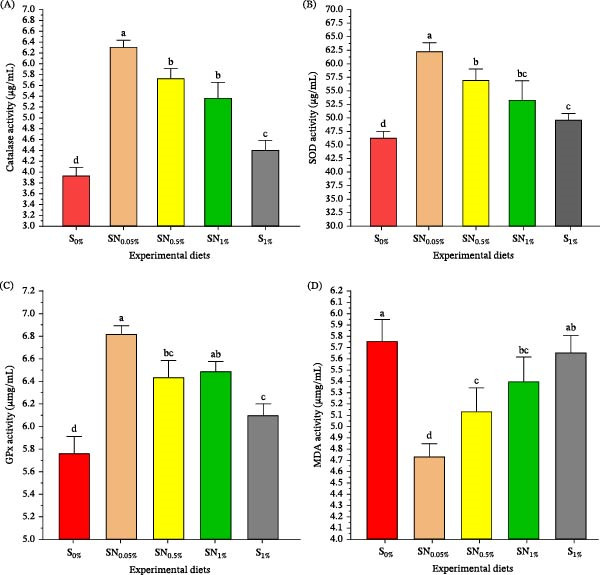
Hepatic antioxidant biomarkers of *M. cephalus* fed the experimental diets. (A) Catalase, (B) superoxide dismutase (SOD), (C) glutathione peroxidase (GPx), and (D) malondialdehyde (MDA). S_0%_: negative control diet contains 0 g *A. platensis*/kg. SN_0.05%_, SN_0.5%_, and SN_1%_: diets enriched with *A. platensis* nanoparticles at concentrations of 0.5, 5, and 10 g/kg of feed, respectively. S_1%_: Positive control diet enriched with non‐nanoform *A. platensis* at a concentration of 10 g/kg. Data are presented as mean ± SD (*n* = 3 replicates per treatment). Means with different letters differ significantly among treatments based on one‐way ANOVA (*p* < 0.001 for catalase; *p* < 0.002 for SOD; *p* < 0.001 for GPx; and *p* < 0.002 for MDA), followed by Duncan’s multiple range test.

The inclusion of *A. platensis* in its nano‐ or non‐nanoform diets considerably enhanced the values of CAT, SOD, GPx, and MDA (Figure 8A–D, respectively). Compared to the control diet (S_0%_), all nanoparticle groups (SN_0.05%_, SN_0.5%_, and SN_1%_) or normal *A. platensis* powder (S_1%_) recorded significantly (*p* ≤ 0.05) improved values for CAT, SOD, and GPx (Figure 8A–C, respectively). The fish fed the SN_0.05%_ group achieved the highest significant CAT, SOD, and GPx activities and the lowest MDA activity, followed by SN_0.5%_ and S_1%_.

## 4. Discussion

### 4.1. Growth Response and Feed Efficiency

The application of algal nanoparticles in aquafeeds has gained increasing attention due to their reported benefits on growth and feed efficiency in fish and crustaceans [14, 19]. However, their effects on mullet culture are still limited. Therefore, this study investigated the impacts of dietary *A. platensis* nanoparticles on *M. cephalus*.

The inclusion of *A. platensis* in the diet, either in conventional (S_1%_) or nano‐form (SN), greatly enhanced the growth performance and feed efficiency compared to the control diet, with the highest growth rate observed in the SN_0.05%_ group. The enhancement is attributed to the higher bioavailability of nano‐*A. platensis* because of the small particle size and large surface area that promote absorption, prolongs circulation time, and improves nutrient utilization [19]. Furthermore, nano‐*A. platensis* may improve intestinal microbial activities that may improve the digestion of complex feed components and absorption of nutrients [38, 46]. Nano‐*A. platensis* is rich in bioactive compounds, including carotenoids, phenolics, essential amino acids, vitamins, and minerals, which have several useful functions [11, 47, 48]. These compounds are responsible for the improvement of feed palatability, which contributes to enhanced feed intake and nutrient efficiency, as suggested by Pakravan et al. [49], resulting in improved feed conversion and protein utilization [50].

The high performance observed in SN‐fed groups supports the hypothesis that nanoparticle delivery improves the transport of *A. platensis* bioactive ingredients across intestinal barriers and into target tissues [19]. Moreover, the antioxidant and anti‐inflammatory compounds present in *A. platensis* play an essential role in reducing oxidative stress and supporting immune competence [9, 51]. In agreement with our results, similar findings have been reported in Nile tilapia, shrimp (*Litopenaeus vannamei*), sturgeon (*Huso huso*), and other species fed nano‐*A. platensis* supplements [19, 25, 51].

### 4.2. Intestinal and Hepatic Histoarchitecture

Intestinal histomorphology is a key determinant of nutrient digestion and absorption in fish [52]. Villus height, width, and muscle thickness directly influence the absorptive surface area, while goblet cells play a critical role in mucosal protection and barrier formation and function [53, 54].

Our results indicate that the dietary inclusion of SN (SN_1%_ and SN_0.05%_) significantly increased the intestinal villi dimensions,muscle thickness, and goblet cell numbers compared to those of the control diet. Nano‐*A. platensis* supplements enhanced the intestinal structural integrity and absorptive capacity, which may enhance nutrient uptake and metabolic activity.

Similar enhancement of the intestinal morphology and immune cell infiltration in different fish species fed *A. platensis* has been reported previously. Rainbow trout, *O. mykiss*, and Nile tilapia fed dietary *A. platensis* also showed an increase in the number of goblet cells, intestinal dimensions, surface area, and intraepithelial lymphocytes [50, 55, 56]. In contrast, reduced villus dimensions and fewer goblet cells were observed in fish fed only the control diet, which may result in impaired nutrient absorption and a reduction of growth.

The liver is the largest gland in the body of fish, which plays a key role in nutrient metabolism and growth‐related endocrine regulation [57]. Fish fed SN diets (SN_0.5%_ and SN_1%_) exhibited reduced lipid accumulation and vascular dilation, indicating improved hepatic health. The strongest hepatoprotective effect was observed at the SN_0.05%_ level. These effects may be due to the high content of carotenoids and phytopigments in *A. platensis*, which provide potent antioxidant protection and decrease lipid peroxidation [9, 58]). Hepatoprotective effects have been reported as well in Nile tilapia and goldfish (*Carassius auratus*) supplemented with *A. platensis* [59, 60].

### 4.3. Serum Biochemical Parameters

In all animals, including fish species, serum biochemical parameters provide valuable indicators of nutritional status, metabolic activity, and organ function [59, 61]. Dietary supplementation with nano‐ and non‐nano *A. platensis* significantly increased serum protein, albumin, and globulin levels, with the highest values observed in the SN_0.05%_ group. This may reflect improved protein synthesis, nutrient utilization, and immune stimulation mediated by the bioactive compounds of *A. platensis* [62–64]. Similar elevations in serum proteins have been reported in Nile tilapia, rainbow trout, and gourami fish fed *A. platensis* or its nano‐form [25, 50, 60, 65–67].

ALT and AST activities are widely used as indicators of liver function. ALT and AST levels were lower in fish fed SN diets than in control, indicating improved liver integrity and metabolic stability [68]. The enzymes ALT and AST play roles in cellular nitrogen metabolism, the oxidation of amino acids, hepatic gluconeogenesis, and liver function. Excessive levels of these enzymes in fish plasma may lead to liver malfunction [68]. Compared to S_0%_ and S_1%_, fish‐fed SN groups (SN_0.05%_, SN_0.5%_, and SN_1%_) exhibited significantly lower AST and ALT activities, indicating the protective impact of *A. platensis* in maintaining the fish liver function in a healthy status and good nutritional futures, as well as circulatory system integrity [69]. Similar hepatoprotective effects of *A. platensis* have been reported in other fish species linked to its carotenoid constituents [11, 70]. In contrast, the inclusion of *A. platensis* at 5%–20% elevated ALT and AST levels in *Nile tilapia* [71].

The urea levels showed no significant difference among all groups. El Gammal et al. [72] reported that *A. platensis* supplementation in tilapia reduced serum urea levels. The NS‐fed mullet had decreased cholesterol and triglyceride levels. This result supports the report of Youssef et al. [56] on Nile tilapia. This suggests a protective function against glycogen storage disease, nephritic syndrome, and liver failure, as reported by Coz‐Rakovac et al. [73]. This effect may be attributed to the lipid‐modulating and antioxidant properties of nano‐*A. platensis*, which inhibit lipid oxidation and support hepatic lipid metabolism [11]. Comparable lipid‐lowering effects have been reported in yellow croakers (*Pseudosciaena crocea*) fed on *Haematococcus pluvialis* [16]. However, Al‐Deriny et al. [50] stated that a low dose of *A. platensis* in tilapia aquafeeds had no effect on blood total cholesterol and triglycerides, while a higher dose of 10% increased them significantly.

### 4.4. Immune Response Parameters

IgM and LYZ are key components of the fish immune system and serve as reliable indicators of immune competence [74, 75]. In this study, dietary inclusion of SN significantly enhanced the IgM concentration and LYZ activity compared with control diets.

The observed immune stimulation may be linked to improved gut health and enhanced intestinal immunity, as well as the immunomodulatory effects of *A. platensis* bioactive components [64]. β‐carotene and phycocyanin, which are found in *A. platensis*, have been shown to stimulate antimicrobial peptide production, inhibit pathogen colonization, enhance the gut microbiota, and enhance phagocytic activity [11, 76]. Enhanced immune responses and enhanced lysosomal activity were also reported in Nile tilapia (*O. niloticus*), coral trout (*Plectropomus leopardus*), common carp, and other marine fish species fed *A. platensis* or its nano‐form [25, 60, 77–80]. Cerezuela et al. [81] reported a similar increase in phagocytic activity in gilthead seabream (*Sparus aurata*) fed *Phaeodactylum tricornutum*. The presence of bioactive compounds such as C‐phycocyanin, vitamin C, and natural antioxidants that inhibit pathogen colonization and stimulate immune responses is the main cause of enhancement of immune functions, as proposed by Zhang et al. [65].

### 4.5. Antioxidant Enzyme Responses

Antioxidant enzymes play a critical role in protecting fish tissues from oxidative damage [76, 82–84]. Dietary SN significantly enhanced the hepatic SOD, CAT, and GPx activities while reducing MDA levels, indicating an improved oxidative balance. These effects are likely due to the strong antioxidant properties of *A. platensis*, which contains carotenoids, phytopigments, and other free‐radical scavengers [65, 85]. Nile tilapia fed nano‐*A. platensis* supplements showed similar antioxidant responses [19, 25]. These results support the protective role of nano‐*A. platensis* supplement against oxidative stress.

## 5. Conclusion

In conclusion, this study suggests that dietary supplementation with *A. platensis* nanoparticles significantly (*P* < 0.05) improved growth performance, feed utilization, intestinal and hepatic histoarchitecture, serum biochemical parameters, immune responses, and antioxidant status in flathead grey mullet (*M. cephalus*). Among the tested treatments, 0.05% nano‐*A. platensis* produced the most beneficial effects. These findings indicate that nano‐formulated *A. platensis* is more effective than its conventional form and may serve as a functional feed additive for mullet culture. Further studies are required to evaluate its long‐term effects and practical applications in different fish species.

## Author Contributions

**Ahmed I. A. Mansour:** conceptualization,methodology, writing – original draft. **Mohamed Ashour:** conceptualization, investigation, formal analysis, writing – original draft, writing – review and editing. **Mohamed M. Mabrouk:** conceptualization, investigation, methodology. **Ahmed F. Abdelhamid:** methodology, investigation. **Othman F. Abdelzaher:** formal analysis, writing – original draft. **Roshmon Thomas Mathew** **, Abdallah Tageldein Mansour, and Yousef Ahmed Alkhamis:** software, visualization, writing – review and editing. **Elsayed S. I. Mohammed:** data curation, software, visualization, writing – review and editing. **Michelyne Haroun:** software, visualization. **Einar Ringø:** data curation, visualization, validation, writing – review and editing, supervision. **Eman Y. Mohammady**: conceptualization, data curation, software, visualization, writing – original draft, writing – review and editing.

## Acknowledgments

The authors would like to thank the National Institute of Oceanography and Fisheries, NIOF, Egypt, as well as Azhar University and King Faisal University, Al‐Ahsa, Saudi Arabia, for their cooperation during this experimental trial.

## Funding

This work was supported by the Deanship of Scientific Research, Vice Presidency for Graduate Studies and Scientific Research, King Faisal University, Saudi Arabia (Grant KFU260184).

## Disclosure

All authors contributed to manuscript revision, read, and approved the submitted version. After using Grammarly software for English language editing, the authors thoroughly reviewed, edited, and verified the entire content manually and take full responsibility for the scientific integrity and accuracy of the publication.

## Ethics Statement

All experiments were approved by the authority of the NIOF Committee for Institutional Care of Aquatic Organisms and Experimental Animals (NIOF‐AQ4‐F‐26‐R‐009).

## Conflicts of Interest

The authors declare no conflicts of interest.

## Data Availability

The authors confirm that all the data and findings in this study are available upon reasonable request from the corresponding author.
